# Nanosized drug delivery strategies in osteosarcoma chemotherapy

**DOI:** 10.1063/5.0137026

**Published:** 2023-02-23

**Authors:** Chenglong Chen, Shidong Wang, Juan Wang, Fangzhou Yao, Xiaodong Tang, Wei Guo

**Affiliations:** 1 Department of Orthopedics, Beijing Jishuitan Hospital, Beijing, People's Republic of China; 2 Musculoskeletal Tumor Center, Peking University People's Hospital, No. 11 Xizhimen South Street, Beijing 100044, People's Republic of China; 3 Wuzhen Laboratory, Jiaxing, People's Republic of China

## Abstract

Despite recent developments worldwide in the therapeutic care of osteosarcoma (OS), the ongoing challenges in overcoming limitations and side effects of chemotherapy drugs warrant new strategies to improve overall patient survival. Spurred by rapid progress in biomedicine, nanobiotechnology, and materials chemistry, chemotherapeutic drug delivery in treatment of OS has become possible in recent years. Here, we review recent advances in the design of drug delivery system, especially for chemotherapeutic drugs in OS, and discuss the relative merits in trials along with future therapeutic options. These advances may pave the way for novel therapies requisite for patients with OS.

## INTRODUCTION

Osteosarcoma (OS), the most common and severe primary malignant bone tumor, is composed of spindle cells that produce osteoid and predominantly initiates in the metaphysis of the long bones with a high occurrence in adolescents and young adults.^1^ It is a highly aggressive tumor and initiates as a monoclonal disease, rapidly developing into a polyclonal one, and is considered one of the most complex tumors in molecular aberration.^2,3^ Since the 1970s, with the introduction of extensive resection and neoadjuvant chemotherapy, the 5-year survival rate of patients with primary OS has increased from 17% to 67%, but is only 20%–30% in patients with concomitant lung metastases.^4,5^ Chemotherapy can eradicate tumor deposits if it is initiated with a low disease burden. Responsiveness of OS to neoadjuvant chemotherapy is a major determinant of clinical outcome for most histologic subtypes, and it is usually defined by a histologic appearance in the resected specimen. The current standard care for OS consists of neoadjuvant chemotherapy (pre-operative), surgical resection, and adjuvant chemotherapy (post-operative).^6^ However, there is no worldwide consensus on a standard chemotherapy approach for OS, and the development of adjuvant chemotherapy has been largely empiric, with the majority of regimens incorporating doxorubicin, cisplatin, and methotrexate or ifosfamide despite the associated drug-related toxicity such as cardiotoxicity and nephrotoxicity.^7,8^

Despite recent novel therapeutic developments for OS, such as immunotherapy, oncolytic virotherapy, and gene therapy, the ongoing challenges in overcoming metastatic OS warrant new approaches to improve overall patient survival, owing to the limited efficacy of chemotherapeutic agents, high level of heterogeneity, and multidrug resistance of OS.^9^ Immunotherapy is highly effective in the treatment of tumors such as hematological malignancies and melanoma, but its therapeutic effect in most solid tumors such as OS is still uncertain.^10^ Oncolytic virotherapy and gene therapy for OS is still in the research stage and cannot yet be used in the clinic. Targeted drugs such as apatinib, cabozantinib, and anlotinib only prolong the disease-free survival of OS patients, but not their overall survival.^3^ Prospects for novel drugs to effectively improve the prognosis of OS patients seemed rather bleak. Furthermore, the requisite high-dose chemotherapy often results in many acute and chronic side effects; hence, identifying novel strategies to amend the disease prognosis and the life expectancy of patients is necessary. Sometimes, a completely new treatment may not achieve immediate results, but it is an innovative and effective solution to improve on an existing treatment; it is time to think outside the lines. Approaches to enhance chemotherapy efficacy by improving delivery methods have yielded promising treatment results in recent years. The first approach attempted to target drug delivery for OS was intra-arterial chemotherapy administration using cisplatin.^11^ This approach relies on the identification of a dose–response curve for OS to achieve high local drug concentration and tumor penetration that could be safely administered in venous infusions.

**FIG. 1.**
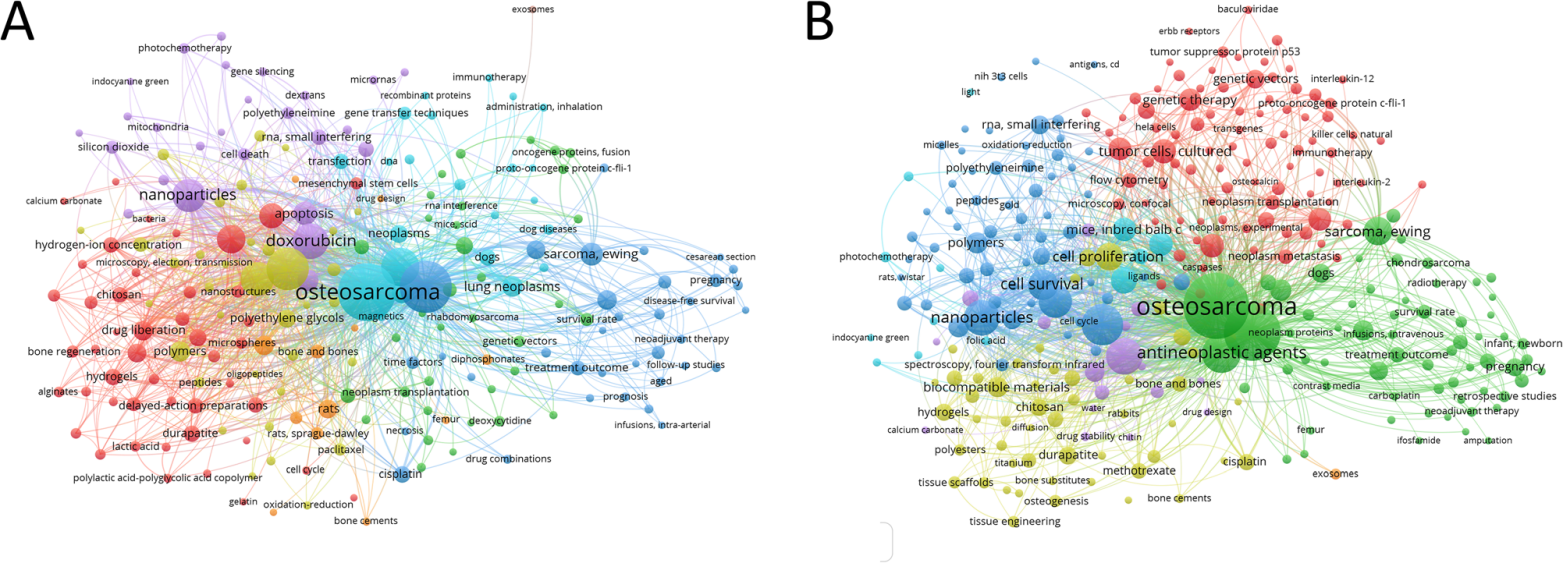
Co-occurrence analysis of global research in PubMed about OS and drug delivery. The size of the points represents the frequency of keywords. (a) Mapping of keywords in OS and delivery system of chemotherapeutic drugs. (b) Mapping of keywords in OS and delivery system of all antineoplastic reagents.

The efficacy of chemotherapy for OS relies on the type of chemotherapy agent, concentration, and absorption efficiency. Meanwhile, the efficiency of drug distribution from plasma to the tumor is affected by some physiologic parameters, such as competitive drug uptake by the liver, excretion of small molecule agents by urine, drug inactivation by binding to proteins, and low stability of the drug in fluids.^12^ Drug delivery and nanomedicine approaches play a pivotal role in modern medicine, ameliorating treatments of conventional drugs due to the ability to modulate the biodistribution and the target site accumulation of chemotherapeutic drugs, thereby reducing their toxicity.^13^ There are several delivery systems for chemotherapeutic drugs that are applied in treating OS, some derived from cellular autocrine liposomal structures, others from synthetic liposomes or macromolecular proteins with or without a targeting ligand.^14^ We searched the keywords in databases to perform a co-occurrence analysis of OS and delivery systems, and the result illustrated in Fig. 1(a) shows that doxorubicin is the most commonly used chemotherapy drug for OS, and apoptosis and liposomes are also hot spots for research in the field. The result illustrated in Fig. 1(b) shows that in addition to doxorubicin and liposomes, cell survival and proliferation, combined modality therapy, chitosan, polyethylene glycols, cisplatin, and hydrogels, are recently being investigated in the area of anti-OS drug delivery. Here, we briefly summarize the advances and rationale in the area of chemotherapy drug delivery technologies for combating OS along with the specific challenges it may face.

## TYPES OF NANOPARTICLES

**FIG. 2.**
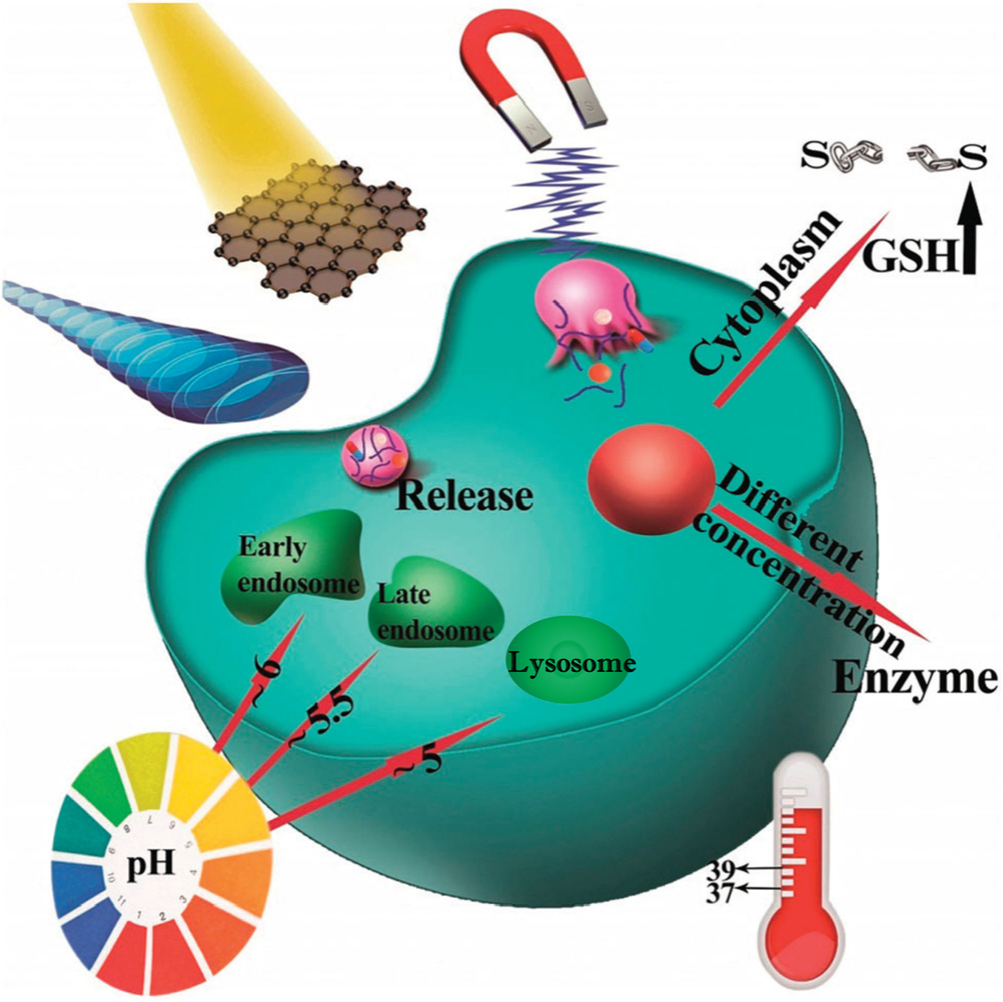
Different physical and chemical stimuli exploited for triggering smart nanoparticles in controlled drug release, namely, pH shift, ultrasound agitation, light irradiation, magnetic induction, redox reaction, enzyme activation, and temperature change. Adapted with permission from Karimi *et al.*, Chem. Soc. Rev. **45**(5), 1457 (2016). Copyright 2016 Copyright Clearance Center, Inc.^15^

Nanosized drug delivery systems can be grossly classified into organic and inorganic carriers.^8^ By exploiting of deep and comprehensive understanding of the cellular and molecular complexity of OS and the convenience of versatile materials, including natural materials, synthetic polymers, lipids, inorganic materials, and biomacromolecule scaffolds, the capability of drug delivery nanocarriers for delivering chemotherapeutics to tumor site has been enormously developed.^12^ Currently, designed drug delivery systems are regularly nanocomposites composed of different types of materials because it is hard to acquire multifunctional nanocarriers from a single nanomaterial. Here, we present some drug delivery systems, combining controlled drug release properties, which can be triggered by various endogenous or exogenous stimulations, as illustrated in Fig. 2.

## LIPOSOMES

Liposomes are spherical vesicles formed by dispersing phospholipids in water, encapsulating part of the aqueous phase, with one or more concentric bilayers of phospholipids inside that allow the encapsulation of drugs with different solubility, ranging from 20 nm to several tens of micrometers in diameter. The physicochemical characteristics of the liposomes, such as surface charge, particle size, and stability, can be adapted to the phospholipid composition. The size, charge, and surface characteristics of liposomes can be easily manipulated by adding other components during the preparation of liposomes or before changing the preparation parameters, altering the half-life period and biodistribution pattern of the drug in the systemic circulation and, thus, improving its pharmacokinetic characteristics. The properties of liposomes make them an ideal delivery vehicle for chemotherapeutic drugs.

Doxil is the first anti-tumor liposome approved by the Food and Drug Administration (FDA) and has been found to have very different pharmacokinetic properties from doxorubicin in clinical trials.^16^ The half-life of Doxil is approximately 90 h, whereas the initial distribution half-life of doxorubicin is only 5 min and the terminal half-life is 20–48 h. The area under the curve (AUC) of Doxil at a dose of 50 mg/m^2^ is 300-folds that of doxorubicin. Subsequent studies have shown that doxorubicin hydrochloride liposomes significantly enhance its bioavailability in tumors. Hu *et al.* developed a biomimetic hybrid nanocarrier loaded with doxorubicin in 2021 by combining liposomes with tumor-derived nanovesicles containing tumor antigens and endogenous danger signals, which can stimulate dendritic cell maturation and elicit subsequent antitumor immune responses for combinational immunochemotherapy.^17^ In this study, doxorubicin-loaded liposomes exhibited synergistic antitumor effects with promoted immunosuppressive effects on tumors and serve as an appealing chemotherapeutic drug delivery system. Similarly, Kleinerman *et al.* reported as early as 32 years ago on the use of monocyte activator muramyl tripeptide phosphatidylethanolamine to activate monocytes *in vivo* by liposome delivery to eradicate residual micrometastases after administration of adjuvant chemotherapy in children with OS.^18^ In combination with current research and clinical understanding, we may be able to combine these properties of liposomes to provide a more robust immune system and internal microenvironment containing well-functional tumoricidal immune cells for OS patients, along with immune-related therapeutic agents, such as IL-1, IL-6, and chemotherapeutic agents, to achieve complete tumor eradication.^10,19^ The effects of liposome doxorubicin combined with ifosfamide on OS patients were evaluated by Huang *et al.*, and the study demonstrated that liposome doxorubicin is exerted more powerful tumoricidal effects to extend overall survival and lower incidences of side effects than conventional doxorubicin plus ifosfamide.^17^ Meanwhile, the liposome delivery system also increased the levels of IFN-γ and IL-10 and reduced the expression of serum TSGF, VEGF, ERBB3, and TNF-α, which are closely associated with OS progression.

However, liposome particles are easily phagocytosed by mononuclear macrophages and are unstable *in vivo.*^20^ Despres *et al.* also reported that immune response can be activated by the surface charge of liposomes, which acts as an immune-activating function against tumors, but is also responsible for their destabilizing effects *in vivo.*^21^ In numerous studies, polyethylene glycol (PEG) in this regard has been used to modify liposomes by steric hindrance stabilization for reducing particle aggregation, thus, enhancing the stability of liposomes *in vivo*, prolonging circulation time, and increasing targeted accumulation at tumor sites.^22^ A phase I clinical trial of advanced OS reported in 2022 demonstrated that combination chemotherapy with pegylated liposomal doxorubicin plus cisplatin reduced cardiac toxicity of doxorubicin and gained an acceptable safety profile and promising clinical benefits.^23^ Despite promising advances in drug delivery, obstacles in balancing the high heterogeneity of OS with systemic circulatory stability are still present. The releases of stable dosage forms of drugs are relatively slow, and hydrophilic drug release is dependent on liposome degradation. Liposomal dosage forms of drugs offer longer systemic circulation time and minimal chemotherapeutic side effects, but at the expense of some efficacy. More studies are needed to strike a balance between obtaining consistently long circulation time and adequate drug release kinetics to achieve improved chemotherapeutic efficacy. Therefore, efforts to develop liposomes that actively trigger the release of chemotherapeutic drugs are of critical importance to advance the clinical application of drug delivery technologies.

## PROTEIN-BASED NANOPARTICLES

Supramolecular nanodrug assembly driven by supramolecular chemistry is becoming an effective strategy for OS treatment. Wang *et al.* reported an engineered proteinaceous nanoensemble that can selectively functionalized as delivering payloads containing doxorubicin to OS cells, exerting a long-acting therapeutic efficacy on OS tumors and remarkable inhibition of pulmonary metastasis along with relieving doxorubicin cardiotoxicity.^24^ By using this nanocarriers based on recombinant proteins extremely prolonged the half-life profile of doxorubicin in nude mice model, and this slow release and targeting effects are both responsible for improving antitumor efficacy and reducing toxicity *in vivo*. Meng *et al.* demonstrated a synergistic therapy by combing chemotherapy with photothermal therapy through a bifunctional polydopamine-modified curcumin-loaded silk fibroin composite in treating OS.^25^ Researchers tested the curcumin loading and controlled release ability of the composite in this study, and the result showed its excellent photothermal properties and a typical pH- and near-infrared-controlled responsive behavior that can release drug rapidly for improving tumor permeation due to the weakly acidic microenvironment of OS and other solid tumors. Meanwhile, an effective inhibition effect on OS cells is also observed. There are obvious advantages of fibroin as a drug delivery system, such as property to controlling sustained drug release, improving drug solubility and stability, along with drug toxicity reduction. However, the problems including the slow degradation of fibroin *in vivo* and its immunogenicity targeted by immune systems remain to be solved.

Similarly, an increasing number of protein-based carriers are being used in delivering chemotherapy drugs, such as lipoprotein, legumin, ferritin, and gelatin. Chen *et al.* designed an oleic acid-modified lipoprotein nanocarrier containing paclitaxel as a pH-sensitive targeting delivery system for tumor treatment.^26^ Lipoprotein nanocarrier showed non-cytotoxicity and pH-dependent drug release properties *in vitro* and targeting anti-tumor activity in tumor-bearing mice. The natural advantage of lipoprotein material, including naturally targeting property, biodegradability, biocompatibility, non-immunogenicity, and long circulation half-life, could be exploited in the future clinical drug treatment of OS. A doxorubicin gelatin microsphere and a doxorubicin polygelatin microsphere were reported in 2019 to be both effectively sustained releasing microspheres that anchored to D-periosteum for eradicating OS cells *in vitro.*^27^ However, this kind of gelatin also has the drawbacks of rapid degradation and low mechanical strength, which needs to be optimized with the properties of other materials in future applications. Azarmi *et al.* established a matrix of parameters to synthesize gelatin-based nanoparticles with different sizes and a narrow size distribution to test the uptake of the nanoparticles by OS cells.^28^ This study also provides theoretical and technical basis for the delivery of chemotherapeutic agents by gelatin-based carriers in OS.

## EXOSOMES

In 1983, Johnstone *et al.* first reported a membranous vesicle released by mature reticulocytes of sheep, which was initially thought to be a carrier of intracellular waste and unwanted proteins, such as transferrin receptors that enter the erythrocyte during maturation, and named the vesicle an exosome.^29^

**FIG. 3.**
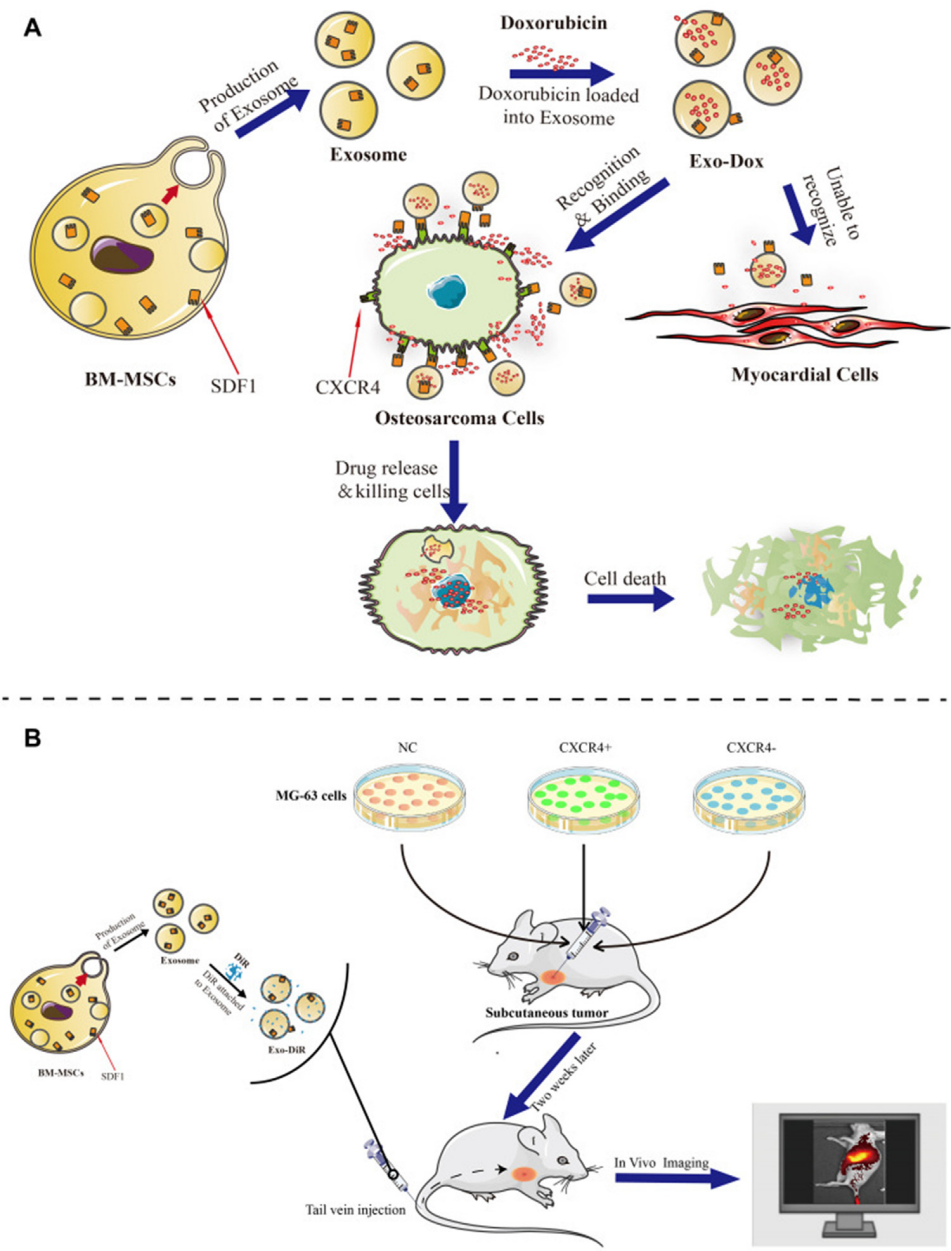
BM-MSC derived exosomes loaded with doxorubicin. The evaluation of the antitumor effect of this system both *in vitro* (a) and *in vivo* (b). Adapted with permission from Wei *et al.*, Int. J. Nanomed. **17**, 35959282 (2022). Copyright 2022 Authors, licensed under a Creative Commons Attribution (CC BY) license.^31^

Exosomes are polycystic macrovesicles of size 40–100 nm in diameter, spontaneously produced and actively secreted by a variety of mammal cells. Exosomes have been recognized as important drug delivery systems and the most commonly used delivery system platform in recent years, carrying a variety of drugs and genetic information, such as proteins and nucleic acids, and travel freely between cell membranes with body fluids. Bone mesenchymal stem cells are frequently used for exosomes extraction in the field of bone tumors. Wang *et al.* used bone marrow mesenchymal stem cells (BM-MSCs) to generate exosomes by sequential extrusion, and successfully encapsulated doxorubicin by an ammonium sulfate gradient method to deliver doxorubicin in OS.^30^ Similarly, Wei *et al.* used mesenchymal stem cell derived exosomes as doxorubicin carrier to treat OS, and result showed an obvious and safe curative effect compared to free doxorubicin.^31^ Figure 3 illustrated the antitumor activity, targeting capability, and mechanism of BM-MSC derived exosomes in OS. Additionally, exosomes from OS and other tumors are responsible for regulating cytokines, either the expression or secretion, and their signaling pathways that regulates tumor growth, angiogenesis, invasion, metastasis, and immune escape.^32^ These functions, which are essential for OS progression and are closely linked to exosomes, may in turn be exploited as targets by using exosomes to inhibit OS growth and metastasis and even drug resistance.^3,33^

## CARBON-BASED NANOCARRIERS

Carbon-based nanocarriers have excellent physicochemical properties including easily modified surfaces, excellent photo-thermal conversion ability, and high adsorption capacity. Ascribable to their merits, numerous carbon-based nanomaterials, such as carbon nanotubes, carbon dots, mesoporous carbon, and graphene oxide, have gained extensive attention and have witnessed a paradigm shift in tumor therapeutics in recent years.^12,34,35^ Augustine *et al.* blocked the TGFβ1-induced OS stem cell formation and the growth of tumors *in vitro* and *in vivo* by using single-walled carbon nanotube nanoparticles.^34^ OS stem cells tend to be resistant to chemotherapy and are closely associated with tumor metastasis, which is the most significant factor in the poor prognosis of OS. Therefore, carbon nanotube represented to be a promising agent for therapeutic applications in OS. Zhang *et al.* designed a carbon-based nanomaterial that increased the effective accumulation of doxorubicin in tumor cell nuclei and tissue and generated massive reactive oxygen species (ROS), thereby restraining tumor proliferation in nude mice model.^36^ Zero-dimensional carbon dots were applied in photothermal therapy under near-infrared irradiation and effectively inhibited OS tumor growth.^37^ For the property of uncontrolled proliferation, tumor cells commonly have the ability to bypass the suicidal process that is a caspase-dependent form of programmed cell death called apoptosis under normal conditions.^4^ Carbon dots was also found to induce the apoptosis of OS cells via the mitochondrial apoptotic signal, thereby inhibiting OS tumor growth and presenting cytotoxicity to 143B cell line.^38^ The same functional capability was found in graphene oxide nanoparticles for inducing apoptosis in wild-type and CRISPR/Cas9-IGF/IGFBP3 knocked-out OS cells that used CRISPR/Cas9 (Clustered Regularly Interspaced Short Palindromic Repeats-associated protein 9) technology for gene-editing.^39^ In this study, Burnett *et al.* discovered that graphene oxide can produce ROS in tumor microenvironment (TME) in a time and concentration-dependent way, which has a significant cytotoxic effect against OS cells. Meanwhile, targeting the IGF1 and IGFBP3 signaling pathway was found to strengthen the cytotoxicity of graphene oxide for restraining tumor progression. However, to date, no studies have been reported on the combined use of graphene oxide and chemotherapeutic drugs in OS, which may be a research direction for the treatment of OS in the future.

## OS-TARGETED DRUG DELIVERY SYSTEM

Targeted drug delivery in OS generally means precise delivery of the intravenously administered chemotherapeutic drugs to the target site by recognizing the properties of some OS or bone tissue. Therefore, it is important to identify the tissue characteristics of OS and find the suitable target for targeted drug delivery. Alendronate is usually used as a target to the bone tissue and is, thus, often seen in the treatment of OS. Morton *et al.* reported an approach that exploits the modularity of Layer-by-Layer (LbL) assembly to produce tissue-specific drug shippers for systemic administration in treating OS.^40^ Because of the strong affinity for intraosseous hydroxyapatite, this alendronate-functionalized poly (acrylic acid) surface gives orthotropic liposome nanoparticles the property of bone targeting; thus, by active targeting of OS xenografts in nude mice with the LbL-targeted doxorubicin liposomes facilitates enhanced, prolonged tumor accumulation and dramatically improved efficacy. By combining with other targeting moieties, alendronate can also form a dual-targeted delivery system. Cluster of differentiation 44 (CD44) is a cell surface receptor highly expressed on OS cells and contributes to doxorubicin chemoresistance, which can be exploited for targeted chemotherapy of OS.^41^ Feng *et al.* developed an efficient OS-targeting liposome system functionalized with a bone and CD44 dual-targeting polymer that conjugated alendronate with a ligand of CD44. Yin *et al.* reported polylactide nanoparticles coated with bone-seeking pamidronate for the targeted treatment of OS in dogs by loading with doxorubicin.^42^ The neat thing about this study is that pamidronate can not only act as a bone-targeting agent to deliver doxorubicin better, but pamidronate itself also is one of the drugs used to treat OS. Therefore, this delivery system can kill two birds with one stone and greatly improve the treatment efficiency. At the same time, an increasing number of targets have been identified and applied for the targeted delivery of OS treatment, such as glucose, mannose, Arg-Gly-Asp, CD133 aptamers, and EGFR aptamers.^43–45^ The combination of these targets with different delivery materials in the future will bring more hope for the precise treatment of OS.

## CELL MEMBRANE-BASED NANOPARTICLES

The cell is the most fundamental unit of life, interacting with its surrounding environments, and it is the outermost layer, consisting of cell membrane, that bears the bulk of this responsibility.^46^ Cell membrane is composed of a mixture of lipids, proteins, and carbohydrates. Compared with conventional strategies that use exogenous materials with the characteristics to be early recognized by the immune system and clearance by the liver and kidney, cell membrane-based strategy exhibits prolonged circulation of the nanoparticles due to its unrecognizable properties by the immune system.^47^ Nanoparticles are coated with cell membranes such as red blood cell (RBC) membrane, immune cell membrane, platelet membrane, and even cancer cell membrane. The first demonstration of cell membrane-based nanoparticles was established by RBC membrane for improving nanoparticle bio-interfacing capacity.^47^ Recently, Fu *et al.* demonstrated a highly stable cancer cell membrane-coated nanoparticles in OS with both physiological and weak acid tumor conditions, which possess homologous tumor targeting capability.^48^ In this report, their strategy significantly inhibited the PI3K/AKT/mTOR pathway in OS, which promotes tumor cell proliferation and cell cycle arrest.^3^ Cell membranes extracted from OS cells and other tumor cells can express “self-markers” and “self-identifying molecules” that can be applied to coat various nanoparticles and provide homologous targeting. A paclitaxel-loaded OS membrane-nanoparticle formulation was used to treat OS, demonstrating a marked ability to increase antitumor effect compared with equivalent doses of free paclitaxel.^49^ Cell membrane-camouflaged nanoparticles can also be used in the diagnosis of tumors. Chen *et al.* used a indocyanine green-loaded and cancer cell membrane-camouflaged nanoparticle system as a theragnostic nanoplatform for homologous-targeting dual-modal imaging and photothermal therapy.^50^ The nanoparticles successfully camouflaged them and reduced the liver and kidney interceptions, and the cell adhesion molecules on the surface of disguised nanoparticles possessed homologous targeted binding to achieve high tumor accumulation. As the emerging cell membrane-based biomimetic nanotechnologies begin to mature, attempts will be made in earnest to translate such platforms to the clinic.

## STIMULI-RESPONSIVE NANOMATERIALS

**FIG. 4.**
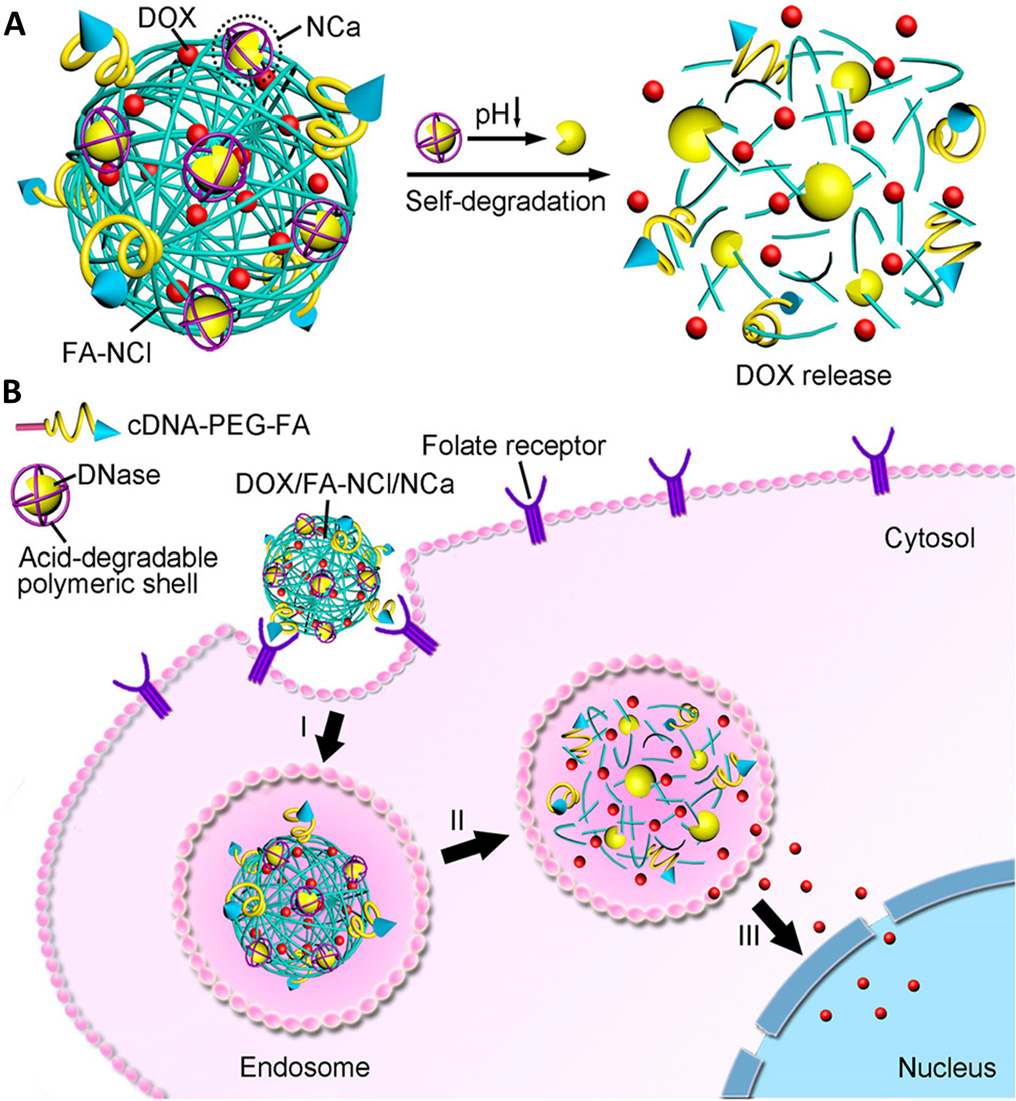
pH-responsive and self-degradable DNA nano-capsule for doxorubicin delivery. (a) pH-triggered self-degradable DNA nano-capsule for doxorubicin release. (b) Schematic illustration of efficient delivery of doxorubicin to nuclei for cancer therapy (I) internalization in endosomes; (II) pH-triggered degradation of the DNA nanoclew for drug release; and (III) accumulation of doxorubicin in cell nuclei. Adapted with permission from Jiang *et al.*, J. Am. Chem. Soc. **136**(42), 14722–14725 (2014). Copyright 2014 Authors, licensed under a Creative Commons Attribution (CC BY) license.^55^

Stimuli-responsive drug delivery has shown promising results in preclinical studies *in vitro* and *in vivo*. The stimuli-responsive drug delivery was first introduced with thermosensitive liposome application for the local release of drugs through hyperthermia in the late 1970s.^51^ Since then, numerous studies have been carried out on the design of stimuli-responsive nanomaterials for drug delivery.^52^ There are two kinds of stimuli-responsive carriers including endogenous stimulated materials and exogenous stimulated ones. The application of endogenous stimuli-response properties is mainly based on the physiological and physicochemical property differences between tumor and normal tissue. Stimuli-responsive nanomaterials are sensitive to specific endogenous stimuli, such as lowered interstitial pH value, high rate of monosaccharide intake, high glutathione concentration, and increased level of certain enzymes such as matrix metalloproteinases.^52^ Rapid proliferation of tumor cells often results in a deficiency of nutrients and oxygen in the tumor microenvironment (TME) and interstitium, which has a lower pH value than the nontumor environment due to the glycolytic metabolism-caused accumulation of acidic metabolites.^8,14^ Equipped with pH-responsive nanocarriers that respond sharply to a subtle change of pH, chemotherapeutic agents can be released more easily in the tumor microenvironment to improve efficacy and reduce damage to normal cells. For instance, Yang *et al.* combined doxorubicin with a fabricated mesoporous ZSM-5 zeolites/chitosan core–shell nanodisk as a delivery system against OS.^53^ This system possesses a highly pH-responsive performance and showed high drug release rate in the weak acidic environment of OS cells *in vitro* and in xenograft nude mice. Figure 4 illustrates a pH-responsive and self-degradable DNA nanocapsule for doxorubicin delivery. Notably, pH-responsive materials also have shared merit of target delivery materials to reduce toxicity to normal cells. Tapeinos *et al.* presented a pH-responsive doxorubicin and cerium dioxide (CeO_2_) delivery system composed with calcium carbonate and collagen type I. It is well known that doxorubicin is the most loaded chemotherapeutics that is developed to target nucleus for antitumor efficacy, and CeO_2_ has its dual ability to scavenge and to generate ROS activated by pH shift of the microenvironment.^54^ Combining the functions of doxorubicin and CeO_2_ in one system offers a powerful therapeutic nanomaterial against OS with reduced heart toxicity compared to free doxorubicin.

**FIG. 5.**
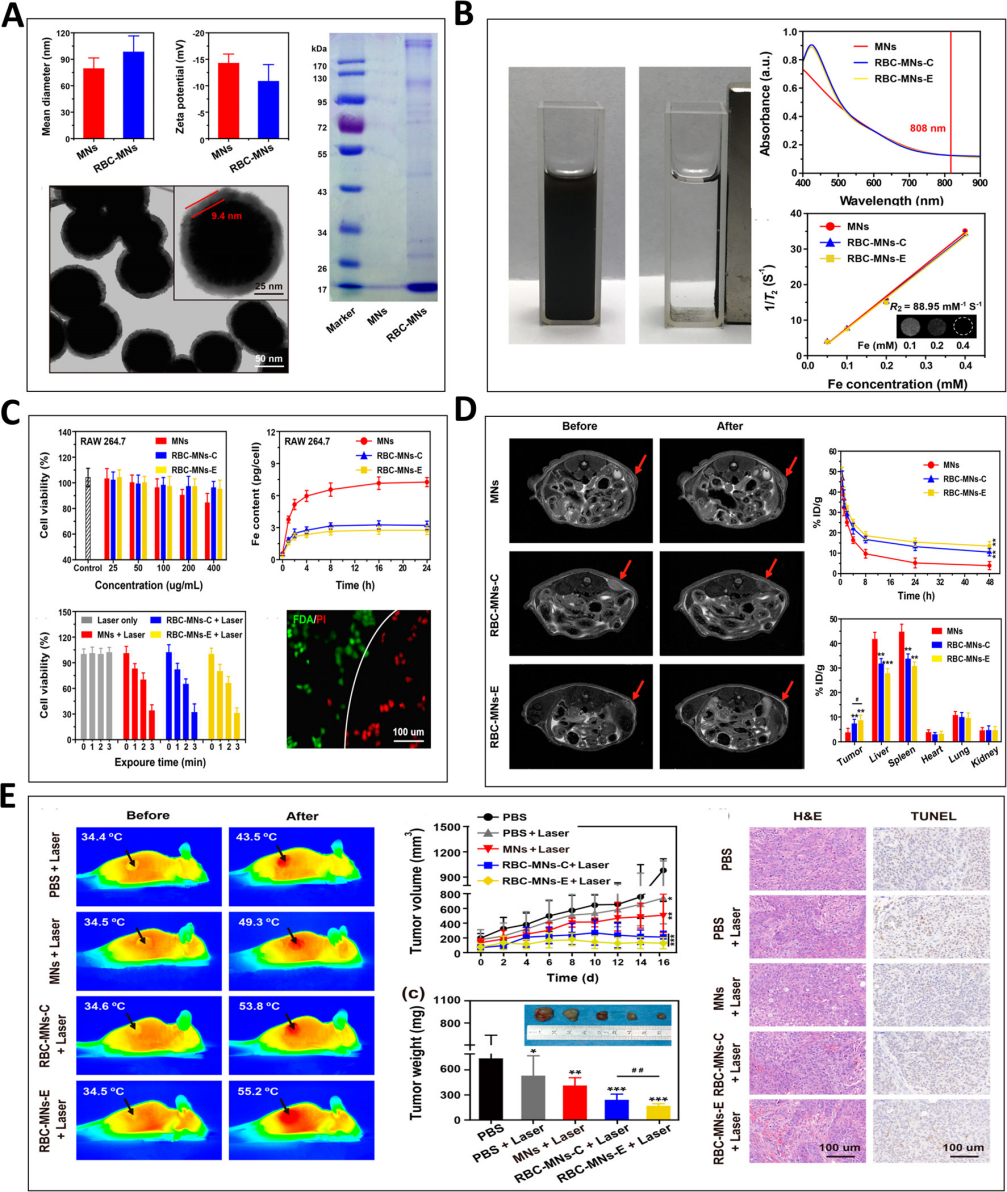
RBC membrane-derived vesicles combined microfluidic technology for *in vivo* tumor MRI and photothermal therapy. (a) Structure characterization of RBC-magnetic nanoparticles. (b) Performance characterization of RBC-magnetic nanoparticles. (c) *In vitro* characterization of the immune escape and photothermal capabilities of RBC-magnetic nanoparticles. (d) *In vivo* tumor MRI and therapy of RBC-magnetic nanoparticles. (e) *In vivo* tumor photothermal therapy with RBC-magnetic nanoparticles. Adapted with permission from Zhou *et al.*, Mater. Sci. Eng.: C **114**, 111006 (2020). Copyright 2020 Elsevier.^63^

The intracellular cytoplasm of cells contains more reductive species than the extracellular matrix.^56^ Meanwhile, there is evidence that the concentration of GSH (glutathione) in tumors is much higher than that in normal tissues, and this provides an application condition of redox-sensitive systems in tumor treatment. For example, Zhang *et al.* developed a polymeric nanomaterial for drug monitoring and synergistic tumor therapy *in vivo* with a GSH-sensitive heterodimeric multifunctional prodrug.^57^ This GSH-responsive prodrug was proficiently loaded into polymeric nanocarrier and co-delivered chemotherapeutic camptothecin and a photosensitizer as synergistic drugs to tumor, leading to a promising curative effect and a visual drug distribution by quantitative PET imaging. In the future of clinical care, visual and monitoring therapies will be essential for tracking and eliminating satellite nodules and metastases of tumors, which are expected to significantly improve the prognosis of patients with OS and other tumors. Figure 5 illustrates a novel monitoring tumor therapy by using red blood cell (RBC) membrane-derived vesicles that modified with magnetic characteristics, and combined microfluidic technology for *in vivo* tumor MRI (magnetic resonance imaging) and photothermal therapy. In addition to the pH- and redox-sensitive nanoparticles, there are also temperature- and enzyme-sensitive materials in the applications of OS treatment. Meanwhile, numerous similar studies provided evidence of spectacular antitumor efficacy of redox-sensitive drug delivery systems applied with alginate nanogels, liposomes, cellulose, hydrogel, chitosan, etc.^58–63^ More strikingly, multi-stimuli responsive drug delivery methods have recently received tremendous attention, which have more comprehensive anti-tumor efficacy. Zhan *et al.*, for instance, demonstrated a degradable multi-modal sensitive poly nanogels combining the properties and merits of different materials and then exhibited thermo-, redox-, and pH- sensitive function for doxorubicin delivery.^64^ On the other hand, multi-stimuli responsive delivery systems often appear as too complicated and many still remain as proofs of concept, which need to be validated both in preclinical and clinical trials.

Exogenous stimuli-responsive drug delivery systems have drawn tremendous attention in recent years, in particular, from the field of oncotherapy. External stimulation generally involved ultrasound, temperature, magnetic field, mechanical forces, light, and electric pulses.^65^ In preclinical studies, the application of ultrasound exposure of microbubbles as a noninvasive and reversible method enhances local area permeability and provides an opportunity for delivery of therapeutic reagents to the target sites of tumors.^66,67^ Some studies have shown that ultrasound, as a physical form of energy, can stimulate the piezoelectric catalytic effect of piezoelectric materials, causing them to produce ROS for killing OS cells. Ultrasound also can be used as a trigger for delivery nanoparticles such as microbubbles to release the drug.^68^ Kou *et al.* reported that the doxorubicin carried in the highly payload capacity of bubbles can be released at the site of OS tissues and induce tumor necrosis with less drug exposure in blood circulation.^66^ A similar conclusion in OS is confirmed by Ueno *et al.* by using a combination of ultrasound and bubble liposome for delivering doxorubicin.^68^

The drug delivery strategies related to light-responsive nanocarriers rely on the electromagnetic radiation property of light, and the electromagnetic spectrum of light used as stimulus often derives from ultraviolet to near infrared wavelengths.^65^ Martella *et al.* synthetized the keratin nanoparticles loaded with paclitaxel and photosensitizer chlorin-e6 for a combination therapy regimen in OS treatment.^66,69^ As a photodynamic and chemo-releasing nanoparticle, this combination regimen delivered paclitaxel efficiently and precisely for inhibiting OS cells in mouse model with ROS enhanced curative effect, decreased general toxicity, and scarce intracellular accumulation. Photo-dynamic therapies are often combined with other noninvasive forms of delivery methods such as thermo-responsive carriers. For example, Ghorbani *et al.* established an injectable light-activated and thermo-responsive methylcellulose hydrogels loaded with sodium humate for local delivery of cisplatin and photothermal ablation in OS.^70^

The controlled release of cisplatin *in vivo* is enabled through the transformation from methylcellulose to gel by light-caused thermal energy, and, at the same time, the light emission causes photothermal ablation of OS cells. Stimuli-responsive nanomaterials have paved the way for a wide range of applications in cancer therapy. The development of stable and innovative drug nanocarriers to meet the clinical needs requires comprehensive understanding of underlying mechanisms after being stimulated in TME and to evaluate the material performances, such as stability, histocompatibility, biotoxicity, bearing efficiency, and the sensitivity to endogenous and exogenous stimulations.^71^

## CHALLENGES AND OUTLOOK

In the current treatment regimen for OS, a combination of chemotherapeutic agents can produce enhanced or synergistic effects and, to some extent, prevent the development of multidrug resistance due to the different mechanisms of action of chemotherapeutic agents.^72,73^ However, chemotherapy treatments cannot solve the inherent disadvantages of traditional chemotherapy drugs, such as poor specificity and undesired pharmacokinetics and biodistribution, which causes serious side effects to normal tissues.^2,10,74^ In addition, combination therapy often has enhanced toxic side effects, and many patients have to terminate the treatment due to intolerance, which greatly limits the clinical application of combination therapy.^5,74–77^ In this condition, novel therapies are crucial for improving survival in OS, especially for patients with metastatic nodes and multifocal relapse. Drug delivery nanocarrier systems are developed to target tumors in combination with other drugs, especially chemotherapeutics in OS, for additive efficacy with less general cytotoxicity.^78,79^

Many delivery systems carry more than one drug to treat tumors. When two antineoplastic drugs are used in combination, especially chemotherapy drugs, the different ratios achieve different effects, which may be synergistic, additive, or even antagonistic.^80–83^ Therefore, it is important to determine the optimal proportions of the two drugs to be used in combination. An ideal carrier nanomaterial tends to be stable, safe, biodegradable, and less toxic, and the dominant factors affecting the properties of nanoparticles are particle size, morphology, surface charge, etc.^84–87^ Nanoparticles with a suitable size can hardly penetrate the blood vessels of normal tissues due to its dense endothelial gap of the microvasculature.^88–90^ On the contrary, it easily and smoothly passes through the vasculature into the OS tissues because of the wide vascular wall space, poor structural integrity, and lack of lymphatic flow in OS and other tumor tissues, playing a certain passive targeting effect.^91,92^ This phenomenon caused by the differences between tumor cells and normal cells is known as enhanced permeability retention effect.^91–94^ Additionally, a suitable particle size is conducive to prolonging the time of circulation in the blood; a particle size less than 10 nm will swiftly extravasate or be cleared by the kidney, while it is easy to cause aggregation in the liver and spleen when the particle size is larger than 200 nm.^95,96^ For now, there is no uniform standard for the optimum size of nanoparticles, but the range of 10–200 nm of nanoparticles is generally believed reasonable.^97,98^ The morphology of nanoparticles affects the circulation time in the blood and the rate of cellular internalization. It is generally accepted that spherical nanoparticles have the shortest circulation time and are the easiest to internalize into cells due to their uniform shape.^99,100^ Furthermore, nanoparticles are more stable when they are slightly negative or positively charged, which can avoid their own aggregation or reduce the interaction with other substances.^101–104^ However, when possessed with a large surface charge, they are easily recognized and cleared by macrophages.

Nanodelivery system of chemotherapeutic drugs has a promising application as it can significantly improve the chemotherapy effect and reduce the adverse reactions in OS. So far, basic studies have been well-explored for achieving the clinical prospects, but still, many of them might never actually enter the clinical practices. Selection of materials according to different patient conditions and types or stages of OS and other tumors needs to be seriously considered. We can select suitable delivery materials, such as materials with low immunogenicity and low damage to the liver and kidney, according to the biochemical values of patients. Genetic test results can be used to screen specific OS targets of the patients and select delivery material and strategy in a personalized manner to reduce the normal cell damage and increase the targeting effect. Various challenges in delivery nanomaterials have been portrayed and need to be addressed, such as how to control the abrupt release of drugs, the ability to cross the biological barriers *in vivo*, specificity and precision, biosafety, histocompatibility, and biodegradability.^52,105–110^ With the deepening research on novel nanocarriers and tumor therapy, it is believed that more efficient drug delivery systems will be applied in clinical settings in the near future, which will fundamentally change the current concept of chemotherapy and greatly improve the prognosis of OS patients.

## Data Availability

Data sharing is not applicable to this article as no new data were created or analyzed in this study.
